# Thalidomide influences growth and vasculogenic mimicry channel formation in melanoma

**DOI:** 10.1186/1756-9966-27-60

**Published:** 2008-11-04

**Authors:** Shiwu Zhang, Man Li, Yanjun Gu, Zhiyong Liu, Shaoyan Xu, Yanfeng Cui, Baocun Sun

**Affiliations:** 1 Department of Pathology, Tianjin Cancer Hospital, Tianjin, Medical University, Tianjin, 300060, PR China; 2 Department of Pathology, Tianjin Dongli Hospital, Dongli District, Tianjin, 300300, PR China; 3 Department of Digestive, Tianjin Second Hopital of Tianjin Medical University, 300211 Tianjin, PR China; 4 Department of Pathology, Tianjin Medical University, 300060 Tianjin, PR China

## Abstract

**Aims:**

To observe the effects of thalidomide on melanoma tumor growth and blood supply patterns in C57 mice.

**Methods:**

Thirty mice inoculated subcutaneously with B16F10 cells were randomly divided into the treatment group and the control group. Thalidomide was administered once a day at a dose of 200 mg/kg for the treatment group starting on the fifth day after inoculation, and an equivalent volume of 0.5% carboxylmethyl cellulose was administered similarly in the control group. The diameter of the tumors was measured daily after inoculation until the mice were sacrificed on the 19th day. The different blood supply patterns were counted after immunohistochemical and PAS histochemical double-Staining. VEGF, NF-κB, PCNA, MMP-2 and MMP-9 expression in tumor tissue was also assessed.

**Results:**

The tumor volume(*P *= 0.019) and the number of vasculogenic mimicry(*P *= 0.03) and mosaic vessels(*P *= 0.004) in the treatment group were significantly decreased compared with the control group. VEGF(*P *= 0.004), NF-κB(*P *= 0.009), PCNA(*P *= 0.002), MMP-2 (*P *= 0.000), MMP-9(*P *= 0.002) protein expression and MMP-2(*P *= 0.000) and MMP-9(*P *= 0.000) mRNA in the treatment group were significantly lower than those in the control groups.

**Conclusion:**

Thalidomide inhibits vasculogenic mimicry channel and mosaic vessels formation in melanoma through the regulation of vasculogenic factors, and it can induce necrosis of melanoma cells, which may be related with the NF-κB signaling pathway.

## Background

Thalidomide was first introduced in the late 1950s for the prevention of morning sickness in pregnant women, but it was withdrawn from the market in the 1960s because of its well-known teratogenicity[1]. Recently thalidomide has been found to have anti-angiogenesis and anti-inflammatory properties, and based on these observations thalidomide has been used as a therapeutic reagent in some malignant tumor including liver cancer, renal cell carcinoma, breast cancer and so on[2]. Many studies also focused on the effects of thalidomide on metastatic melanoma. The mechanism of thalidomide against melanoma maybe attribute to its anti-angiogenic activity. However, the detail mechanism is still unclear. Except of endothelium-dependent vessels(EVs), there are vasculogenic mimicry (VM) channels and mosaic vessels(MVs) in melanoma, which constitute the blood supply pattern for some high-grade malignant tumor [3,4]. In this study we established a mouse model bearing melanoma tumors and examined the effect of thalidomide on tumor growth and angiogenesis patterns.

In 1999, Manitotis *et a l*[5] described a novel paravasculars tumor blood supply pattern named VM. The VM channels are lined by tumor cells in the absence of endothelial cells and fibroblasts, and red blood cells appear in these channels. There are periodic acid-Schiff (PAS)-positive extracelluar matrix (ECM) surrounding these channels or covering on their surface, which may play important roles in VM formation. Based on microarray analysis, highly aggressive melanoma and less aggressive melanoma differ in the expression of about 210 genes including some genes associated with the phenotypes of endothelial and hematopoietic stem cells [6]. Tumor cells with embryonic-like phenotype have more plasticity, and can mimic endothelial cells and participate in the formation of VM channels [7,8]. The expression and secretion of matrix metalloproteinases (MMPs) also play an important role in ECM remodeling. MMP-2 and MMP-9 are members of MMPs family and they can functionally degrade collagen IV in ECM, a step essential for tumor invasion and metastasis[9]. Moreover, MMP-9 generates growth-proliferating signals and regulates tumor cells proliferation[10]. The mechanism of thalidomide's anti-angiogenic effect may be associated with its teratogenecity involving with MMPs secretion [11,12]. This paper was focused on thalidomide influencing different blood supply patterns formation on animal experiment, and the related molecular mechanism is also introduced.

## Materials and methods

### Drug and Animals

Thalidomide was kindly provided by Changzhou Corp. It was dissolved in 0.5% sodium carboxylmethyl cellulose (CMC) at a final concentration of 20 mg/ml. Animals Inbred, clean, black, 6–8 week old C57BL/6 mice were obtained from Institute of Hematology and Blood Disease Hospital affiliated Chinese Academy of Medical Science(License number: SCXK(Jin)2004-0001). They arrived in Animal Centre of Tianjin Cancer Hospital one week before the experiment and were bred under SPF. This study was approved by the Medical University of Tianjin's Animal Welfare Committee.

### Model of melanoma and tumor experiment in C57 mice

C57BL/6 mice (n = 30) were injected subcutaneously with 2 × 10^6 ^murine melanoma B16F10 cells into the lower left groin. Thirty mice were randomly divided into treatment group and the control group, with 15 mice in the treatment group and 15 mice in the control group. Thalidomide was dissolved in 0.5% sodium carboxyl methylcellulose. Five days after B16F10 melanoma inoculation, 0.2 ml thalidomide (200 mg/kg/d) was administered via interperitoneal injection once a day in the treatment group and an equivalent volume of 0.5% CMC alone was administered in the control group. After administration, there was not obviously symptom of disorder of digestive tract in the mice of the treatment group. The length and width of the subcutaneous tumors were measured using a caliper every day. The tumor size was calculated according to the following formula: Tumor volume (cm^3^) = (length × width^2^)/2. The tumor growth curve was drown based on tumor size. On the 19^th ^day after inoculation, all mice were sacrificed, and the tumor tissues were harvested. Part of the tumor without necrosis were collected and stored at -80°C and the remainder of the tumors were fixed with formalin and embedded in paraffin.

### Real-time PCR to detect MMP-2 and MMP-9 mRNA expression levels

Total RNA was extracted with Trizol reagent according to the manufacturer's instructions. 1% agarose electrophoresis and detection of OD260/OD280 ratio were performed to identify the integration and purity of isolated RNA. Complementary DNA (cDNA) was synthesized and amplified from total RNA using the Access real time PCR system (TaKaRa). The primer sequences used for matrix metalloproteinase (MMP)-2(GeneID: 17390) detection were 5'- GATAACCTGGATGCCGTCGTG -3' (sense) and 5'- CTTCACGCTCTTGAGACTTTGGTTC -3' (anti-sense). The primer sequences used for MMP-9 (GeneID: 17395) detection were 5'-GCCCTGGAACTCACACGACA-3' (sense) and 5'-TTGGAAACTCACACGCCAGAAG-3' (anti-sense). The primers used to amplify β-actin were 5'-CATCCGTAAAGACCTCTATGCCAAC-3' (sense) and 5'-ATGGAGCCACCGATCCACA-3' (antisense). The resultant cDNA products of MMP-2, MMP-9 and β-actin were 109, 86 and 174 base pairs, respectively. Real time PCR products were analyzed with the Gene AMP PCR System 5700 Sequence Detector and the C_T _values were evaluated. The CT value (the cycle number at which the fluorescence crosses the threshold) was determined and 2^ΔCT ^where ΔCT = ΔCT_MMPs_-ΔCT_β-actin _was defined as the relative quantity of the amplified fragment. Every sample was tested in triplicate and the mean value was used. The products of real-time PCR were validated with 1.5% agarose electrophoresis.

### Histopathological examination and Immunohistochemical staining

For histopathology studies, tumor tissues were cut in the center to obtain the largest section and indicate the information of the whole tumor. They were then fixed in 10% buffered formalin, dehydrated, and embedded in paraffin using routine methods. For immunohistochemical staining, formalin-fixed, paraffin-embedded tissue was sectioned and dried overnight at 65°C and deparaffinized in xylene. The sections were rehydrated through graded alcohols into water. Endogenous peroxidase was blocked with 3% hydrogen peroxide in 50% methanol for 10 min at room temperature. After rehydrating, the sections were washed with PBS and then pretreated with citrate buffer (0.01 M citric acid, pH 6.0) for 20 min at 100°C in a microwave oven. After rinsing with PBS, slides were incubated overnight at 4°C with primary polyclonal antibodies (rabbit anti-human, mouse and rat), including the antibodies against vascular endothelial growth factor(VEGF), proliferating cell nuclear antigen(PCNA) (Boster Biological technology Ltd, Wuhan, China, dilution 1:150), nuclear factor-κB (NF-κB, Upstate, New York, USA, dilution 1:100), MMP-2 (BA0596, Boster Biological technology Ltd, Wuhan, China, dilution 1:100), and MMP-9 (BAO573, Boster Biological technology Ltd, Wuhan, China, dilution 1:100). The sections were then washed with PBS and incubated with the second antibody for 30 min at 37°C. The sections were incubated with secondary antibody (Non-Biotin HRP detection system, Zhongshan goden bridge biotechnology CO., Ltd, Beijing, China) for 30 min at 37°C after the PBS washes. Visualization was performed using a DAB Kit(DC 10, Boster Biological technology Ltd, Wuhan, China) under microscope. The nuclei were counterstained with hematoxylin, followed by dehydration and coverslip mounting. Appropriate positive and negative controls without primary antibody were included.

### Immunohistochemical and PAS histochemical double-Staining methods

Antibodies used in this study were mouse monoclonal anti-CD34 (Sigma Chemical Co., St. Louis, Mo, USA) and mouse monoclonal anti-HMB45(BM0093, Boster Biological technology Ltd, Wuhan, China, dilution), which were used at dilutions: 1:400. After immunohistochemical staining, Sections were exposure to 1% sodium periodate for 10 min. The sections were then rinsed with distilled water for 5 min and incubated with periodic acid-Schiff (PAS) for 15 min. Finally, all of the sections were counterstained with hematoxylin, dehydrated and mounted. Normal human stomach mucous membrane was the positive control.

### Quantification of determination of the positive ratio for VEGF, NF-κB, PCNA, MMP-2, MMP-9 and microvessel patterns count

When stained for VEGF, NF-κB, MMP-2 and MMP-9, tumor cells with brown cytoplasm were considered positive, and when stained for PCNA, tumor cells with brown nuclei were considered positive. We observed 10 fields per section at 400× magnification, and positive cell numbers were counted in 100 random melanoma cells in every field. The mean percentage of positive cells was used to determine the expression of the proteins in a section. EVs, MVs and VM channels were also counted. All these counts were blindly performed in at least 3 randomly chosen sections from each mouse. The mean value of each type of microvessel in 10 fields was the final outcome.

### Statistical analysis

Statistical software SPSS 10.0 (Chicago, Illinois) was used in the analysis. A *P *value less than 0.05 was considered statistically significant. Two-way ANOVA was performed to evaluate melanoma growth of two groups. Counts of distinguished blood vessels in the treatment group and the control group were evaluated with one-way ANOVA. Differences of protein and mRNA expressions between two groups were compared using an unpaired *t *test.

## Results

### Effects of thalidomide on growth of melanoma

**Figure 1:**
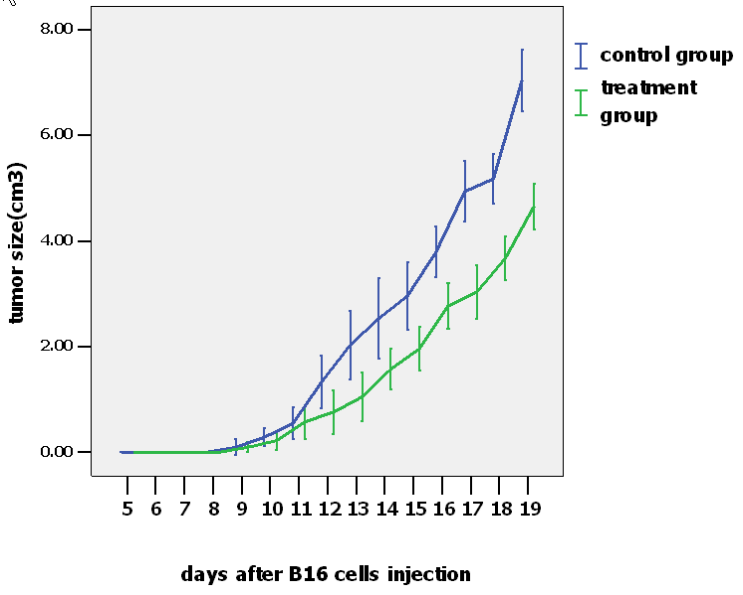
**Effects of thalidomide on B16F10 melanoma growth.** Error bars represent standard deviations for the experimental and the control groups. In the early stages of the experiment, there are no significant differences in tumor volume, but from day 12 to day 19 the tumor volume in the treatment group showed a clear reduction compared with the control group. There is statistical significance between these two groups when the mice are sacrificed (*P *= 0.019).

**Figure 2:**
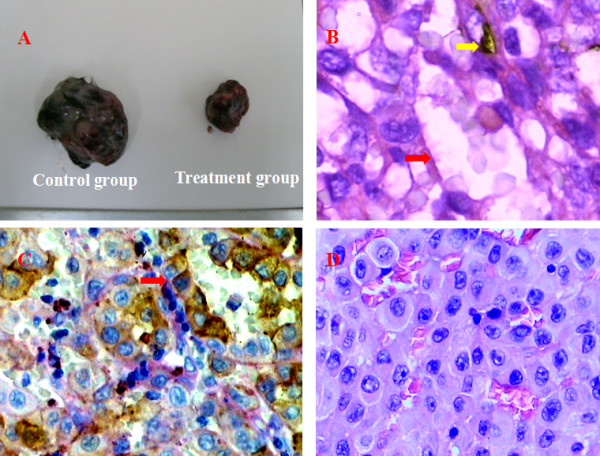
**Different microcirculation patterns in melanoma tissue of murine xenograft model.** A. Tumor volume in the control group was larger than in the treatment group, suggesting thalidomide inhibits tumor growth. B. VM channel (red arrow) was formed by tumor cells and there were red cells in the center of the channels. PAS-positive substances lined these channels and formed basement membrane-like structure. Yellow arrow indicated EVs presenting in the same field with VM. Endothelial cells were stained as brown by immunohistochemical staining for CD 34, CD34 and PAS double-Staining, 1000×. C. Red arrow showed a VM channel that was lined by brown melanoma cells as well as pink PAS-positive substances. Melanoma cells were identified by HMB45 antibody. HMB45 and PAS double-Staining, 400×. D. In the control group, mitosis was present and VM channels were more numerous under low-magnify field. H&E, 200×.

Ninth days after melanoma cell injection, engrafted tumors were palpated on the mouse groin areas and detected tumors were removed. Soft globular tumors were observed and there were numerous network vessels on the tumor surfaces. Some melanoma cells invaded into the skeletal muscle and showed a spindle configuration. The engrafted melanomas in the thalidomide treatment group grew slower than those in the control group (Figure 1). Fourteen days after the thalidomide administration, the average size of the tumor in the treatment group was 6.912 cm^3^, while in the control group it was 4.164 cm^3^. The tumor volume in the treatment group was decreased compared with the control group and there was statistical significance between these two groups (*P *= 0.019) after the mice were sacrificed. In the early stages of the experiment, there were no significant differences in tumor volume, but from day 12 to day 19 the tumor volume in the treatment group showed a obvious reduction compared with the control group (Figure 1 and Figure 2A). There were large areas of necrosis in tumor tissues of the treatment group, while necrosis was not obvious in the control group. Numerous melanoma cells of the treatment group were characterized as cell degeneration including vacuoles within the cytoplasm and nuclei. However, in the control group, there are only some tumor cell degeneration in the centre of tumor mass.

### Results of immunohistochemical and PAS histochemical double-Staining methods

We performed PAS staining and staining for the endothelial cell markers CD34. CD34 is a marker of endothelial cells and the base membrane is positive for PAS. CD34 immunohistochemical and PAS histochemical double-staining was used to distinguish VM, MVs and EVs. HMB45 immunohistochemical and PAS histochemical double-Staining was used to identify the origin of tumor cells lining VM channels and the structure of VM as well. Cells lining VM channels were negative for CD34 and positive for HMB45 confirmed that cells around the channels were not composed of endothelium but melnaoma cells (Figure 2B and 2C). Some channels lined with both CD34-positive/PAS positive and HMB45-positive/PAS positive cells were MVs. Red cells in the centre of VM channels indicated that they may be connected with EVs. Under microscope with 400× magnification, EVs, MVs and VM channels were counted and the three microcirculation patterns could co-exist in melanoma(Figure 2D). The number of VM(*P *= 0.03) and MVs(*P *= 0.004) in the treatment group were decreased compared with the control group. There were fewer EVs in the treatment group compared with the control group, but the difference was not statistically significant (*P *= 0.068) (Table 1).

**Table 1:** Comparison of the mean number of three microcirculation patterns between the treatment group and the control group ($\bar{x}$ ± SD)

	Control (n = 15)	Experimental (n = 15)	t	*P*
VM channels	1.86 ± 0.89	0.58 ± 0.37	3.08	0.030
MVs	0.90 ± 0.45	0.33 ± 0.19	3.54	0.004
Endothelium- dependent vessel	2.22 ± 1.52	0.54 ± 0.37	2.44	0.068

* SD: Standard Deviation

### Inhibition of VEGF, NF-κB, PCNA, MMP-2 and MMP-9 expression by thalidomide

**Figure 3:**
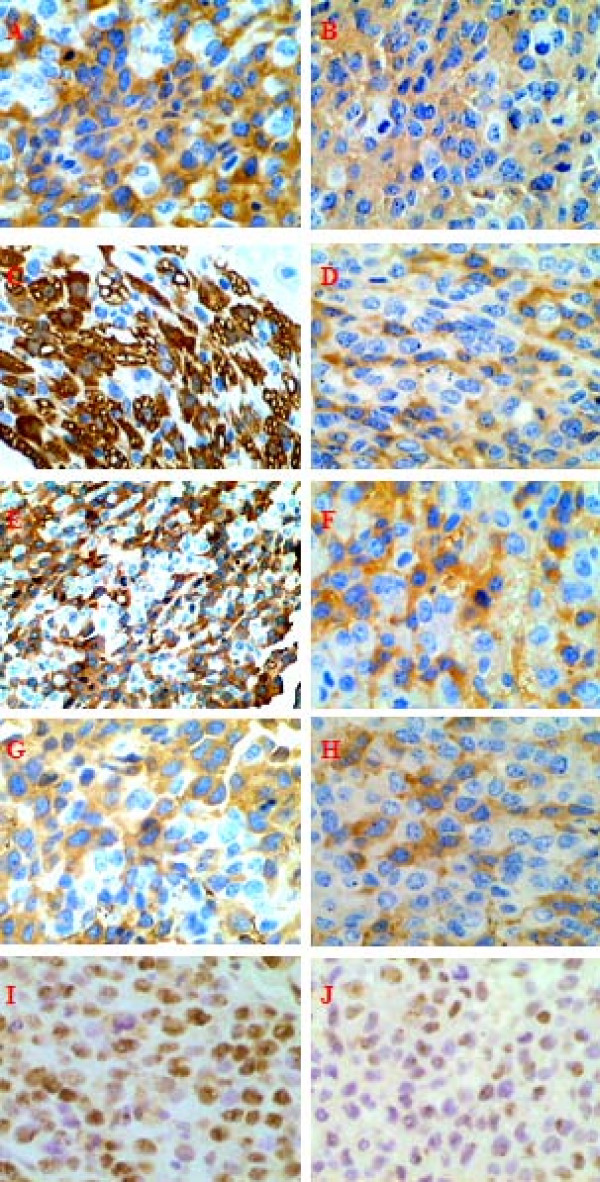
**Immunohistochemical staining results of VEGF, NF-κB, MMP-2, MMP-9, and PCNA.** VEGF, NF-κB, PCNA, MMP-2, MMP-9 protein expression including the number and staining intensity of positive cells in the treatment group were significantly lower than those in the control groups. A. VEGF expression in the control group. Tumor cells showed brown cytoplasmic staining. IHC, 400×. B. VEGF expression in the treatment group. IHC, 400×. C. NF-κB expression in the control group. Tumor cells showed brown cytoplasmic staining. The immune reaction in the nuclear was rare in this study. IHC, 400×. D. NF-κB expression in the treatment group. IHC, 400×. E. MMP-2 expression in the control group. Tumor cells showed brown cytoplasmic staining. IHC, 200×. F. MMP-2 expression in the treatment group. IHC, 200×. G. MMP-9 expression in the control group. Tumor cells showed brown cytoplasmic staining. IHC, 200×. H. MMP-9 expression in the treatment group. IHC, 200×. I. PCNA expression in the control group. Tumor cells showed brown nuclear staining. IHC, 200×. J: PCNA expression in the treatment group. IHC, 200×.

Both the treatment group and the control group contained positive tumor cells. The positive positions of tumor cells for VEGF, NF-κB, MMP-2 and MMP-9 staining were located in the cytoplasm and for PCNA in the nucleus. VEGF(*P *= 0.004), NF-κB(*P *= 0.009), PCNA(*P *= 0.002), MMP-2(*P *= 0.000), and MMP-9(*P *= 0.002) expression in the treatment group was significantly decreased compared with the control group, including overall number and staining intensity of the positive cells (Figure 3 and Table 2). The number of tumor cells positive for PCNA staining in the treatment group was less than observed in the control group. Results of PCNA immunohistochemical staining indicated that the tumor cells in the treatment group had a lower proliferative ability than that in the control group.

**Table 2:** Comparison of the mean percentage of positive cells for VEGF, NF-κB, PCNA, MMP-2 and MMP-9 expression between the treatment group and the control group ($\bar{x}$ ± SD)

	Control (n = 15)	Experimental (n = 15)	t	*P*
VEGF	18.34 ± 2.32	13.67 ± 2.51	3.47	0.004
NF-κB	17.92 ± 4.45	12.21 ± 2.84	3.05	0.009
MMP-2	24.06 ± 3.92	10.93 ± 3.35	2.36	0.000
MMP-9	19.32 ± 3.66	10.46 ± 3.76	2.31	0.002
PCNA	80.94 ± 4.90	67.55 ± 7.75	2.18	0.002

* SD: Standard Deviation

### Results of Real-time PCR

Real time PCR demonstrated that the expression of MMP-2 mRNA in the treatment group was decreased compared with the control group, which was similar to MMP-2, MMP-9 protein expression. The C_T _value of MMP-2 and MMP-9 in the control group was lower than that in the treatment group. The results of real time PCR show that thalidomide could down-regulate MMP-2 and MMP-9 mRNA expression, suggesting that thalidomide can inhibit tumor growth via down-regulation of the MMP-2 and MMP-9 mRNA level(Table 3). There were statistical significance for MMP-2(*P *= 0.000) and MMP-9(*P *= 0.000) mRNA levels between the treatment group and the control group.

**Table 3:** Real time PCR results for C_T _value of MMP-2 and MMP-9 in the treatment and the control groups ($\bar{x}$ ± SD)

	Experiment (n = 15)	Control (n = 15)	t	*P*
MMP-2	0.0122 ± 0.0014	0.0719 ± 0.0021	6.254	= 0.000
MMP-9	0.0025 ± 0.0006	0.0198 ± 0.0018	11.263	= 0.000

* SD: Standard Deviation

## Discussion

Malignant melanoma is one kind of the most aggressive form for skin malignant tumor. Malignant melanoma has proven to be highly resistant to conventional chemotherapy and operation is still the main therapeutic method for melanoma. Patients with malignant melanoma generally have a high risk of recurrence and a short survival time[13]. Thalidomide was withdrawn from the market due to its teratogenecity, but in recent years its use has been focused on its anti-angiogenic property[1,14-16]. Thalidomide has been used in phase II trials as a treatment for solid cancers such as renal cell cancer and hepatocellular carcinoma where it has shown significant anti-tumor effects [17,18]. It has been proposed that the teratogenic property of thalidomide involves in the production of reactive oxygen species (ROS), leading to subsequent DNA damage[5]. Thalidomide generates ROS that affect differentiation of murine embryonic cells during vasculogenesis and angiogenesis in the embryo [19,20]. When thalidomide-induced ROS formation is inhibited, the anti-angiogenesis properties of thalidomide will be reduced.

It had been believed that EVs are the only microcirculation mechanism present in tumors for a long time, so endothelium are the target of the traditional anti-angiogenesis treatment for solid tumors [21,22]. However, VM, an endothelium-independent microcirculation pattern, has been demonstrated to exist in many malignant tumor types. These findings suggest that the VM channel should be an additional target in an anti-angiogenesis strategy to treat solid tumors [23-27]. A result of cDNA microarray from Seftor EA et al [7,8] confirmed that highly aggressive melanoma cells with VM expressed more embryonic-like genes, while they were absent in poorly aggressive ones. Hence thalidomide targeting at embryo angiogenesis and anti-vasculogenesis may exert its influence on VM channels formation though inhibiting embryonic-like genotype.

VM channels, MVs and EVs coexist in melanoma and contribute to the melanoma blood supply in the B16 melanoma model[28]. The presence of VM channels, and MVs not only meet the need for oxygen and nutrients required for tumor growth but also enhance tumor metastasis. In this study VM channels and MVs were significantly decreased in the treatment group compared with the control group. This phenomenon may be related to the anti-angiogenesis and anti-VM formation property of thalidomide. One feature of VM formation is that the tumor cells join together to form an extracellular matrix-rich network contributing to a paravascular system that coexists with endothelium-lined vessels. In addition, MMP family members, especially MMP-2 and MMP-9, play important roles in endothelium-dependent vessels and VM channel formation[29]. Collagen IV is a major constituent of basement membrane ECM and a major function of MMP-2 and MMP-9 is to degrade collagen IV resulting in promotion of angiogenesis. Furthermore, MMP-9 can induce VEGF to be secreted into ECM resulting in enhanced tumor angiogenesis[8,30].

In this report, tumor growth in the treatment group was significantly decreased compared with the control group. Results of our study agreed with the idea that inhibition of tumor growth by thalidomide was dependent on the blockage of angiogenesis. It has been reported that thalidomide inhibits angiogenesis by interrupting processes mediated by vascular endothelial growth factor (VEGF) and basic fibroblast growth factor (bFGF)[31]. Thalidomide has also been described as an inhibitor of TNF alpha, probably by enhancing the degradation of TNF alpha mRNA[32]. VEGF, known as a vascular permeability factor that enhances vascular permeability and promotes new vessel growth, was thought to be one of the factors responsible for angiogenesis[33]. In this study, VEGF expression in the treatment group was decreased compared with that in the control group, suggesting that the mechanism of thalidomide inhibiting tumor angiogenesis maybe related with reducing VEGF expression. Furthermore, NF-κB has been found to have anti-apoptosis, pro-angiogenesis, and pro-metastasis properties. Thalidomide inhibits H_2_O_2_-induced NF-κB activation[34]. In this study, NF-κB and PCNA expression were also decreased in the thalidomide treatment group compared with those in the control group. These data suggest that NF-κB also play important role in thalidomide preventing tumor cell proliferation, angiogenesis.

Three factors govern the formation of functional and patterned microcirculation channels by VM: (1) plasticity of highly malignant tumor cells, (2) remodeling of the ECM, and (3) the connection of the VM channel and host blood vessels to acquire a blood supply from the host tissue[24]. Matrix metalloproteinases (MMPs) can cleave one or several constituents of the extracellular matrix (ECM) [30]. MMPs can degrade ECM which facilitates vascular formation and tumorigenesis. A key step of VM formation is that MMPs secreted by tumor cells digest ECM and remodelled of ECM. The remodeling of ECM has been correlated with MMP overexpression. Highly aggressive melanoma cells have the ability to secrete MMPs and degrade ECM in vitro[8]. In our experiment, the expression of MMP-2 and MMP-9 was decreased in the treatment group compared with the control group. Thalidomide may inhibit MMP-2 and MMP-9 expression and thereby inhibit VM channel formation.

In this report, we demonstrated that the effect of thalidomide on the microcirculation patterns in the B16F10 mouse melanoma involved inhibition of microcirculation formation and protein expression. In this experiment, The dose of thalidomide we used at 200 mg/kg/day is comparable to 4 mg/kg/day in humans that is a safe level without serious side effects[35]. Thalidomide promoted B16F10 melanoma cell necrosis and inhibited VEGF, NF-κB, PCNA, MMP-2 and MMP-9 expression. EVs, MVs and VM channels contributed to the microcirculation patterns in the B16F10 melanoma model described in this report. The presence of the three microcirculation patterns not only meets the need for oxygen and nutrients required for tumor growth but also enhance metastasis. MMP family members, especially MMP-2 and MMP-9, play important roles in EVs, MVs and VM channel formations. These data provide a basis for considering thalidomide for use in the treatment of solid tumors with VM.

## Abbreviations

VM: vasculogenic mimicry; EV: endothelium-dependent vessel; MV: mosaic vessel; MMP: matrix metalloproteinase; ECM: extracellular matrix; VEGF: vascular endothelial growth factor; PCNA: proliferating cell nuclear antigen; ECM: extracellular matrix; CMC: carboxylmethyl cellulose; NF-κB: nuclear factor-κB

## Competing interests

The authors declare that they have no competing interests.

## Authors' contributions

ZS carried out the animal experiment, participated in the design of the study. LM participated the animal experiment and carried out morphological observation. GY carried out the immunohistochemical staining. LZ performed the statistical analysis. XS participated in the study design and coordination. CY carried out the data collection and helped to draft the manuscript. SB carried out the design of the study. All authors read and approved the final manuscript.
